# Rate of biological invasions is lower in coastal marine protected areas

**DOI:** 10.1038/srep33013

**Published:** 2016-09-09

**Authors:** A. Ardura, F. Juanes, S. Planes, E. Garcia-Vazquez

**Affiliations:** 1 Laboratoire d’Excellence «CORAIL», USR3278-CRIOBE-CNRS-EPHE-UPVD, Université de Perpignan-CBETM, 58 rue Paul Alduy, 66860 Perpignan Cedex, France; 2 Department of Biology, University of Victoria, Victoria, BC, V8W 3N5, Canada; 3 Department of Functional Biology, University of Oviedo. C/ Julian Claveria s/n. 33006-Oviedo, Spain

## Abstract

Marine biological invasions threaten biodiversity worldwide. Here we explore how Marine Protected areas, by reducing human use of the coast, confer resilience against the introduction of non-indigenous species (NIS), using two very different Pacific islands as case studies for developing and testing mathematical models. We quantified NIS vectors and promoters on Vancouver (Canada) and Moorea (French Polynesia) islands, sampled and barcoded NIS, and tested models at different spatial scales with different types of interaction among vectors and between marine protection and NIS frequency. In our results NIS were negatively correlated with the dimension of the protected areas and the intensity of the protection. Small to medium geographical scale protection seemed to be efficient against NIS introductions. The likely benefit of MPAs was by exclusion of aquaculture, principally in Canada. These results emphasize the importance of marine protected areas for biodiversity conservation, and suggest that small or medium protected zones would confer efficient protection against NIS introduction.

There are many potential pathways by which exotic species may be introduced. In the marine realm the main vectors of biological invasions are linked to aquaculture and shipping development^1^,^2^,^3^,^4^,^5^,^6^. The process of biological invasions is complex and involves either intrinsic features of the invasive organisms^7^,^8^ and/or opportunistic colonization of degraded environments^9^ and empty niches^10^. Although there is discrepancy in patterns in different studies^11^, species richness of marine communities is expected to be negatively correlated with invasion success^10^. Examples of stronger invasion occurring where there is more native biodiversity can be rectified by considering the scale of the study and the approach (experimental versus observational)^12^. In addition to native biodiversity, habitat status also seems important for biological invasions. In an extensive review, MacDougall and Turkington^13^ found that habitat degradation is a promoter of biological invasions.

The benefits of marine protected areas (MPAs hereafter) have been shown for both ecosystem structure and functioning^14^. MPAs can act as population sources of exploited species, thereby contributing to improved fisheries sustainability^15^. They provide shelter for endangered organisms^16^ and preserve trophic chains^17^. If they are located at adequate distances from each other, improved connectivity helps to enhance effective population size of keystone and singular species^18^. Larger MPAs are generally desired^19^, although the amount of area to be protected for efficient protection of natural resources is a controversial question^20^. Even small MPAs are beneficial for biodiversity, especially if they form a network at relatively short distances^15^,^21^. On the other hand, both MPA size and age are keys for ensuring efficient protection of biodiversity, as the effects of marine reserves on biodiversity are positively correlated with the duration of the protection scheme^22^.

The benefits of MPAs for biodiversity have been demonstrated in many studies, but their social acceptance is difficult in some instances. MPAs restrict the use of marine resources and may be negatively perceived by local stakeholders^23^,^24^,^25^. Incongruence between management intent and fisheries permitted within MPAs has been found in some cases^26^. If local fishers attempt to use fishing resources illegally within a reserve to maximize their economic payoffs, more efforts may be required to maintain reserve effectiveness^27^. Social issues involved in MPA planning and management are hence important for reconciling local development and environmental protection^28^,^29^. At the same time, the public sees marine invasive species as a risk for ecosystem services, including recreation and human health, and supports the investment in programs against biological invasions^30^. Demonstrating that biological invasions are better controlled by MPAs could contribute to improving social acceptance of marine protection. However, the effect of MPAs in NIS control has been poorly studied to date. In some of the few experimental investigations of this topic, Byers^31^,^32^,^33^ found negative interactions between native exploited species and NIS molluscs in protected areas of the northeast Pacific. Some studies suggest that MPAs may contribute to NIS control. For example, resilient native communities with less NIS than non-protected areas have been found in coral reef reserves^34^; similarly, a lower proportion of NIS has been observed inside compared to outside MPAs in Moorea Island^5^. However, in a meta-analysis Burfeind *et al*.^35^ suggested similar performance of NIS outside and inside of marine reserves, and concluded that there were too few data available about the effects of spatial protection on NIS. The present study aims at filling this gap.

Different mechanisms and factors promote biological invasions^2^,^3^,^4^,^11^,^13^. The MPA would likely control NIS through a combination of them. Fewer NIS vectors are expected to occur within MPAs (e.g., less or no aquaculture, restrictions to maritime traffic), thus the occasions for NIS to arrive inside the MPA are scarcer than in unprotected areas. On the other hand, environmental stressors may be lower in MPAs and NIS have less empty habitat for recruiting than in unprotected spaces. Native biodiversity is also expected to be higher within MPAs^14^,^36^. Assuming that there is biotic resistance from the native community that scales with its diversity, new species will find it more difficult to invade a healthy community than an impoverished one. We expect many possible combinations of environmental status and local biodiversity in different MPAs. Hence for a similar level of protection NIS number may still vary in different regions and circumstances.

In order to obtain generalizable conclusions, here we use two Pacific islands of very different size, type of human uses and climate as models, and treat them equally for analysis of NIS impacts and protection scaling. Islands are especially affected by biological invasions, thus provide *a priori* a good geographical setting for our study^37^. Molluscs are chosen as the taxonomic model group because they contain a large proportion of recognized marine invasive species. We apply a genetic Barcoding approach to avoid taxonomic incertitude in polymorphic and cryptic species^38^.

## Results

**Figure 1:**
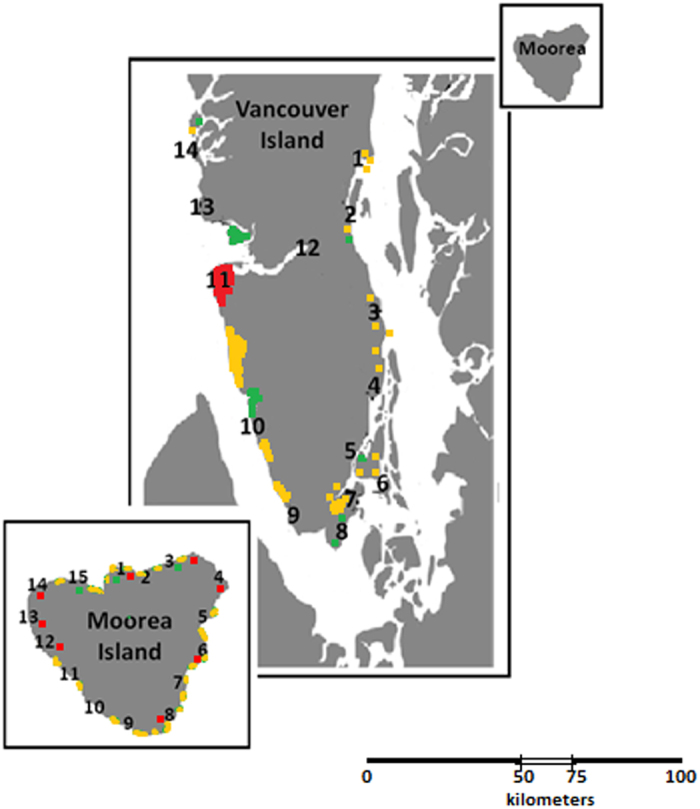
Map of the sampling sites and protected areas in the two Pacific islands considered. Moorea Island is represented at the same scale as Vancouver Island in the small square above right, and at x10 scale in the square below left. Red, yellow and green colors mark areas with high, medium and low protective status respectively, as it is represented in official websites of each island. Lack of color means no spatial protection. Manually drawn by Alba Ardura with the software Microsoft Paint within Microsoft Office 2013. They were generated with OpenStreetMap licensed under CC BY-SA (www.openstreetmap.org/copyright). The license terms can be found on the following link: http://creativecommons.org/licenses/by-sa/2.0/ **Vancouver Island**: 1. Cortes Island, 2. Fanny Bay, 3. Nanoose Bay, 4. Ladysmith, 5. Crofton, 6. Portland Island, 7. Sidney, 8. Victoria, 9. Sooke, 10. China Beach, 11. Bamfield, 12. Port Alberni, 13. Salmon Beach, 14. Long Beach. **Moorea Island**: 1. Entre 2 Baies, 2. Pao-Pao, 3. Maharepa, 4. Temae, 5. Vaiare, 6. Fareahu, 7. Afareaitu, 8. Maatea, 9. Atiha, 10. Vaianae, 11. Haapiti, 12. Tiki, 13. Hauru, 14. Tiahura, 15. Papetoai, 16. Opunohu.

The mollusc species and samples per site obtained from Moorea Island were described in Ardura *et al*.^5^. The same data for Vancouver Island are shown in Supplementary Table 1. Briefly, on Moorea Island a total of 1487 individuals were sampled from 16 sites corresponding to 26 species (3 Bivalvia and 23 Gastropoda). On Vancouver Island a total of 1049 individuals were found in the 14 sites considered (Fig. 1). They corresponded to 32 species: 12 Bivalvia, 18 Gastropoda and 2 Polyplacopohora (chitons) (Supplementary Table 1). The COI and 16S rDNA sequences that identify these species were submitted to GenBank (http://www.ncbi.nlm.nih.gov/genbank/), where they are available for COI and 16S rDNA, respectively, with the accession numbers KC732781-KC732805, KP06789-KP06818, KT970491-KT970498; and KP06819-KP06834, KT970487-KT970490.

As expected from the environmental conditions, human uses and management policies, the intensity of each NIS vector was highly variable between sites (Table 1). The incidence of aquaculture was higher on Vancouver Island, especially on the east coast, than in Moorea. The east coast of Vancouver Island had less MPAs than the west coast. Substrate was more artificial in Moorea than in Vancouver Island (3.63 versus 2.93 of average substrate modification on a 1–5 Likert scale for Moorea and Vancouver islands respectively). The observed maritime traffic, however, was similar in the two islands, slightly more intense in Vancouver (2.9 on average on a Likert scale) than in Moorea (2.56). As a NIS vector the local traffic intensity value could be underestimated on Vancouver Island as we measured it (counting ships from the sampling points), because harbors are much bigger there and receive many more cargo tons than harbors of Moorea.

The proportion of NIS individuals (Table 1) was 2.6% and 5.1% on Moorea and Vancouver islands respectively. The number of NIS species was 5 in Moorea (19.9% of the total number of species) and 6 (18.8%) in Vancouver Island: 6 Gastropoda and 5 Bivalvia. The five NIS bivalves are included in the ISSG list of global invasive species, International Union of Conservation of Nature (http://www.issg.org/database/welcome/, accessed June 2016): *Mytilus edulis*, *M. galloprovincialis*, *Ruditapes philippinarum*, *Crassostrea gigas* (all on Vancouver Island) and *Saccostrea cucullata* (in Moorea). One of the gastropods, *Batillaria atramentaria* (in Vancouver Island), is recognized as a global invasive species on that list. *Nuttalia obscurata* is a recognized invader in the Northeast Pacific (e.g. Byers 2002). The four exotic gastropods found in Moorea (*Drupa albolabris*, *Littoraria glabrata*, *Thais tissoti*, *Nerita tessellata*), and *Nucella lapillus* in Vancouver Island, are not considered invasive but are not native to Polynesia or the northeast Pacific respectively.

The NIS were not homogeneously distributed on the two islands (Table 1). Aquaculture was positively and significantly correlated with NIS (r = 0.673, P = 0.00005 for 28 d.f., significant after Bonferroni correction). At the local level spatial protective status (PS) was negatively correlated with a lower proportion of NIS (r = −0.601, P = 0.0004 for 28 d.f., significant after Bonferroni correction). In order to check if the duration of the protective status (MPA age) and the status itself could be considered independently for NIS prevention we calculated the corresponding linear correlations. For MPA age the result was not significant (r = 0.236, P = 0.209), but the status alone was significant after Bonferroni correction (r = −0.543, P = 0.0019). Thus the two variables combined in a multiplicative way, here called PS, improved the level of significance.

No significant correlations between NIS and any other variable were found after Bonferroni correction (data not shown). We found no significant correlations between local and 10-km, or between local and 60-km PS (r = 0.417 and r = −0.008 respectively, both not significant after Bonferroni correction). This could be a consequence of a patchy and irregular distribution of MPAs in these islands (Fig. 1).

**Figure 2:**
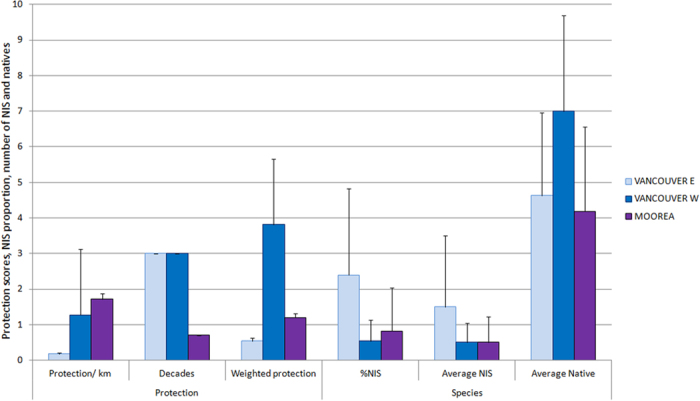
Marine protective status (as Protection) and species distribution (as Species) at the regional scale in each coast considered. Moorea Island (MOOREA) is labeled in purple; east and west coasts of Vancouver Island (VANCOUVER E and VANCOUVER W) are labeled in light and deep blue respectively. Protection includes: coastal protective status as *protection/km*, on a Likert scale; duration of protection as *decades*, and their product as *Weighted protection*. Species include % of NIS over total number of species as *%NIS*, mean number of NIS over the sampling sites in each region as *Average NIS*, and mean number of native species per site as *Average native*. Vertical lines with caps show standard deviation.

The negative relationship between NIS frequency and coastal PS was also clear at the regional level (Fig. 2). The area that has been moderately protected for a longer time, the west coast of Vancouver Island, and the island with the larger proportion of coast protected (although for less time), Moorea, exhibited less NIS and lower proportion of NIS individuals than the least protected east coast of Vancouver Island. On the other hand, the average number of native species was higher on the west coast of Vancouver Island. The correlation between the PS and the number of native species per site was only marginally significant when status was considered at a large (60 km) spatial scale (r = 0.372, 28 d.f., P = 0.042), and not significant for other scales. Thus, the proportion of NIS in reserves was lower because there were fewer NIS not more native species.

Regarding diversity estimates, as expected, diversity estimates were highly correlated to each other (r = 0.698, P = 1.79E-05). Species richness was highly correlated with the number of native species (r = 0.901, P = 1.15E-11), as was the Simpson index (r = 0.635, P = 0.000163). Local PS was marginally negatively correlated with species richness (r = −0.520, P = 0.003, not significant after Bonferroni correction with 15 variables) and the Simpson index (r = −0.368, P = 0.039). Correlations between NIS proportion and diversity indices were not statistically significant (r = 0.243, P = 0.196 and r = 0.136, P = 0.472 for correlation of NIS with species richness and Simpson index respectively). However, significant autocorrelation was found between each of the indices and NIS (Durbin-Watson statistic of 0.711, P = 2.7902E-05 and 0.762, P = 6.865E-05 for species richness and Simpson index respectively), thus diversity estimates were not included in further global analysis of the whole dataset.

**Figure 3:**
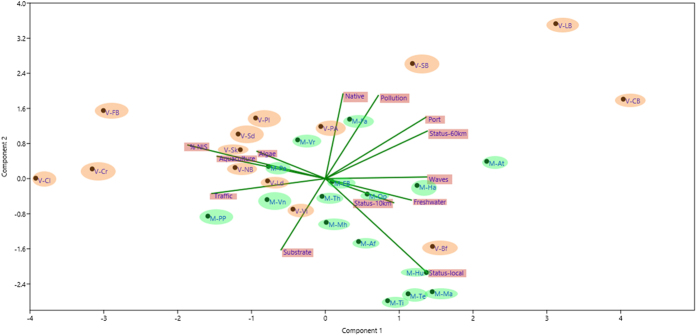
Scatter plot obtained from Principal Component Analysis of the sites and variables considered in this study. The diagonals (in green) represent the variables analyzed (red squares) and are proportional in length to the relative contribution of each variable to the total variance. Site acronyms (M for Moorea, green ellipses; V for Vancouver, orange ellipses): PP, Pao-Pao; Pa, Papetoai; Th, Tiahura; Fa, Faarehau; Vn, Vai’ane; Vr, Vai’are; Ti, Tiki; At, Atiha; Hu, Hauru; Mh, Maharepa, Te, Temae; Ma, Maatea; EB, Entre-2-Baies; Op, Opunohu; Af, Afareitou; CI, Cortes Island; Cr, Crofton; FB, Fanny Bay; NB, Nanoose Bay; PI, Portland Island; Ld, Ladysmith; Sd, Sydney; Vi, Victoria; Sk, Sooke; CB, China Beach; Bf, Bamfield; PA, Port Alberni; SB, Salmon Beach; LB, Long Beach.

The principal component analysis (PCA) allowed us to identify two outlier sites (Fig. 3): China Beach and Long Beach from Vancouver Island. These two sites are anomalous because their distance to the nearest port is much longer than that of any other sampling point, thus this factor (distance to port) exhibited a discontinuous distribution if they were included. They were removed from our models in further analyses. Consistently with the correlations explained above, NIS proportion and local PS (Status-local in Fig. 3) were placed in quadrants 4 and 2 respectively and exhibited the longest diagonals, indicating opposite trends and maximum contribution to the dataset variance. Aquaculture was almost in the same position as NIS. In Fig. 3 it can be observed that despite very different conditions in the two islands (Table 1), the sites were not grouped by island but interspersed, with many sites from Moorea and Vancouver distributed in similar zones of the plot. Exceptions were four sites from Moorea (Hauru, Maatea, Temae, Tiki) and three sites from Vancouver Island (Cortes Island, Crofton, Fanny Beach), grouped by islands and located apart from the rest of the sites. Those four sites from Moorea had the maximum level of local PS, no NIS, and at the same time low number of native species, while the three sites from Vancouver Island had minimum level of protection and high proportion of NIS. The four principal components in the dataset accounted for 65% of the variance (Table 2), with NIS, local PS, 10-km PS and the number of native species providing the maximum load in PC1, PC2, PC3 and PC4 respectively.

The Multiple Linear Regression model (MLR) with NIS as the dependent variable and excluding outliers, provided a multiple R value of 0.913 (R^2^ = 0.834, R^2^ adjusted  = 0.702). The ANOVA was highly significant (F = 6.298, d.f._1_ = 12, d.f._2_ = 15, P = 0.0006). Significant independent variables were local PS with a negative sign, and aquaculture with a positive sign (Table 3, left). The ordinary least square regression of NIS and local PS was highly significant (r = −0.605, t = −3.873, P = 0.0006, permutation P = 0.0016), as well as that of NIS and aquaculture (r = 0.671, t = 4.617, P = 0.0001, permutation P = 0.0012). The Durbin-Watson statistic was not significant for these variables (1.683, P = 0.203 for NIS-aquaculture and 1.834, P = 0.322 for NIS-PS) or any other, thus autocorrelations in the dataset could be discarded.

The Generalized Linear Model (GLM) provided similar results (Table 3, right). Only aquaculture and local PS had significant regression slopes with NIS. Local PS and NIS were significantly correlated, and aquaculture had much greater statistical significance; whereas with the MLR it was the opposite. With the GLM the effect of maritime traffic was marginally significant (P = 0.05), while with the MLR the marginally significant variable was substrate artificiality (P = 0.07).

Since this was a correlational study and the two case studies were geographically distant and ecologically different, their combination could generate analytical artifacts. There are many predictor variables that do not vary independently among regions as well as a completely different suite of species and different species inventories (that determine accuracy of NIS identification). In order to alleviate this problem the models were tested again in subsets of samples composed of different regional combinations. We made different groups of samples: [west Vancouver Island + Moorea], [east Vancouver Island + Moorea], [Vancouver Island], and [Moorea] (Table 4). The results obtained from the MLR and GLM were generally consistent in the four subsets and coincided largely with the results obtained from the whole dataset. MLR was globally significant in [east Vancouver Island + Moorea] (ANOVA, F  =  6.085, P  =  0.0016), and was marginally statistically significant for [Moorea] (F = 3.169, P = 0.049). Significant correlations between NIS and local PS were found in all the subsets. In addition, in [east Vancouver Island + Moorea] and in [Vancouver Island] statistical significance was found for aquaculture, and for substrate artificiality in [east Vancouver Island + Moorea]. In the GLM the local PS was statistically significant in all the subsets (Table 4, columns at right). Aquaculture was significant in the subsets that included east Vancouver Island, which is logical because aquaculture occurs almost exclusively in the samples of that region. The only discrepancy between the GLM and the MLR found in these subsets was the absence of statistical significance for substrate artificiality in [east Vancouver Island + Moorea] under the GLM. These results emphasize the importance of local protective status for NIS prevention because it was significantly related with NIS, always negatively, in all the subsets.

The correlation between native biodiversity (measured as species richness or Simpson’s index) and NIS was not significant in any subset (data not shown).

## Discussion

The robust statistical support for a negative relationship between MPA and NIS in islands of very different ecological and environmental conditions suggests that spatial protection prevents the settlement of exotic species. Boudouresque and Verlaque^39^ pointed out that introduced species may undermine the protection efforts of MPAs because diminishing the use of protected spaces would enhance both native and invasive species simultaneously. However, it did not seem the case in the studied islands since NIS were not equally abundant in protected and unprotected areas. With sample sizes of approx. 100 individuals per site in our sampling design, scarce species may have been overlooked, as well as early NIS arrivals (still in very low proportion in a site). However the number would be reasonable for detecting established invasions.

Enhanced native diversity could prevent NIS from settling^10^,^12^, thus we expected higher native diversity within MPAs. However, although the number of native species was marginally correlated with protective status at the regional level (60 km coast), we did not find higher diversity in MPAs at that scale, either species richness or Simpson’s, when the two islands were considered together. Possibly the case studies considered here, with relatively low diversity since they were focused only on sessile molluscs, are not the best examples for investigating the attractive idea of species richness and evenness as factors of biotic resistance against invasions.

The main mechanism acting against NIS that was revealed in our case studies could be reduced NIS vectors near MPAs (principally aquaculture in Canada), thus preventing massive and repeated NIS arrivals. Lessening opportunities for introduction of new species (by lowering propagule pressure) was probably one of the main mechanisms behind MPAs carrying less NIS. Although in Canada fishing restrictions might not be properly implemented inside MPAs^26^, it seems that aquaculture was the major driver of NIS in this analysis; so perhaps Canada’s MPAs are effective against aquaculture. In addition to preventing from introduction, a combination of other factors was likely responsible for such protection. In pandemic invasions vectors seem to act synergistically^2^. In our model the NIS vectors/promoters were not significantly correlated to each other; moreover, some of them like local maritime traffic and ports did not correlate significantly with NIS. Only in one subset, [east Vancouver Island + Moorea], did artificial substrate reach statistical significance (Table 4). Interestingly we have seen that globally recognized invader species were abundant in Vancouver Island (six out of 11 NIS), more affected by aquaculture and slightly more by shipping than Moorea. In contrast, in Moorea only the promoter (artificial substrate) but not the vectors was more intense than on Vancouver Island. Accordingly only one out of five NIS found in Moorea (*Saccostrea cucullata*) was recognized as a global invader. The vectors would explain the quantity of NIS that reaches a coast (our model suggests that MPAs prevent NIS arrival), and the synergy or multiplication of vectors would promote invasions^2^, probably together with habitat degradation, native biodiversity erosion, and other factors. The severity of biological invasions would depend on complex interactions between vectors and local environmental and diversity status, and less on the number of NIS arriving.

The spatial scale at which MPAs operate may be relevant. Spatial autocorrelation may play a role here. Although not statistically significant for most of the variables analyzed here, at the small scale sites in one studied region will share many environmental attributes (in addition to protective status and the other variables considered in this study). On the other hand, NIS and MPAs were negatively correlated at the local scale in this study, but the relationship disappeared at a larger scale (60 km). This scale-dependency could be related to the coastline’s patchiness. Protected areas alternate with unregulated zones where NIS can be introduced more frequently. Moreover the movement of small boats, rarely forbidden in MPAs, could disperse NIS at any distance along the coast^40^, contributing to erosion of the possible effect of marine protection at the regional scale. Our results again suggest the importance of treating MPAs at small scales, already proposed after discovering short-distance dispersal of some key species^21^.

The spatial scale recognized in this study, based on sessile molluscs, falls within the lower range of the dispersal capacity of most marine organisms; for example, many reef fish exhibit dispersal ranges of 10 to 200 km^15^,^41^, and invertebrates 10–100 km^15^. A NIS established at a point could disperse beyond the largest distance considered in this study (around 60 km). If at the new location there was spatial protection and the local biodiversity was rich and healthy, the NIS would probably have little chance to become an invader^17^. From the point of view of invasions, many small MPAs would be efficient in controlling NIS in a region. On the other hand, in this work we have focused on coastal MPAs, that is, coastal protected areas. From our data we think that inferring the effect of subtidal protection could be somewhat speculative. We expect that reducing the uses of subtidal zones (e.g., less maritime traffic, fishing vessels and fishing gear; no floating aquaculture cages etc.) would reduce the number of NIS entering a zone, and also the NIS in the intertidal.

Finally, this study analysed two very different case-studies for dimension, environment and latitude, and found MPAs can protect against NIS invasions. The similar result across different regions suggests that MPAs may more generally protect against NIS. Currently marine spatial planning typically does not consider NIS. Our study suggests spatial planning can incorporate NIS to help protect local biodiversity.

## Methods

### Study sites and their protected areas

The two islands were represented in the maps of Fig. 1.

The island of Moorea (French Polynesia; 17°32′S 149°50′W), in the tropical Pacific Ocean, was recently studied for inventorying NIS molluscs and inferring their main carrier vectors. Maritime traffic was determined as the principal factor for introduction of NIS^12^. Aquaculture was limited to a single shrimp farm (*Penaeus stylirostris*), a small pearl oyster farm (*Pinctada margaritifera*) and some old and marginal attempts to culture grouper (*Epinephelus merra*) and other fish.

Vancouver Island (Northeast Pacific, 49°30′N 125°30′W, temperate climate) is characterized by substantial aquaculture activity. There is intense shellfish culture of mussels, clams and oysters, both native and imported species including invasive species such as the Mediterranean mussel *Mytilus galloprovincialis*, the manila clam *Ruditapes philippinarum* and the Asian Pacific oyster *Crassostrea gigas* (http://www.dfo-mpo.gc.ca/aquaculture/sector-secteur/species-especes/, accessed September 2015). Aquaculture was identified as the main vector of introduction of exotic mussels^13^. The source and impact of other exotic molluscs have not been investigated as exhaustively to date.

The protected areas of Moorea and Vancouver islands were found respectively in http://www.commune-moorea.pf/les-services-municipaux/environement/p-g-e-m-plan-de-gestion-de-lespace-maritime/ and http://www.env.gov.bc.ca/bcparks/explore/map.html (both accessed in September 2015). In the indicated websites there was a full description of the types of protection and the age of each protected area, as well as the detailed uses permitted under each type of protection. In brief, the density, mean size and age of the MPAs were different between the two islands. On Moorea Island, spatial planning was implemented only about 10 years ago (2004). About one third of the coastline was dedicated to 8 MPAs, and 2 additional areas were designed as regulated fisheries. On Vancouver Island the situation was different. The protected areas were older (30 years on average, with some already initiated in the 1950 s), and the protected coastline varied between the two sides of the island. There were 3 types of protection frameworks, from more to less protection with: national parks, ecological reserves and regional parks. Overall, protection covered roughly 10% and 3% of the west and east coast respectively. Aquaculture was concentrated in the east coast (less protected one), and some species such as manila clam and Pacific oyster were cultivated in the Strait of Georgia, where mussel farms were also concentrated. Naturalized populations of *Crassostrea gigas* occurred in the area^42^.

### Sampling

Sampling was carried out in 2011–2012 from rocky beaches in each location. As described in Ardura *et al*.^5^, 100 molluscs were sampled from approximately 200 m^2^ at each site proportional to the abundance of each species as identified *de visu* (at random within species). Samples were taken haphazardly from the different elements in the area (rocks, boulders, concrete walls, artificial materials…) in a balanced manner, roughly proportional to the relative surface covered by each material. We also searched under the algae –where they were present. The reason was the intertidal environment can be patchy, and the different species may have different microhabitat preferences. The same sampling scheme was followed in the two islands. The intertidal range (upper to lower) was covered.

In total 16 and 14 sites were sampled from Moorea and Vancouver islands respectively. The names, locations and main ecological characteristics of the sampling sites can be found in Ardura *et al*.^5^ and Crego-Prieto *et al*.^6^ for Moorea and Vancouver islands respectively. In addition we took into account substrate artificiality as a sign of habitat degradation, which is a facilitator or promoter of biological invasions (Ardura *et al*.^12^). Briefly, these characteristics were measured as follows: distance to the closest freshwater source and to the closest pollution source, in m; wave exposure as the % surface directly impacted by waves during the intertidal time; algae coverage as % surface covered with algae; substrate artificiality as % of anthropogenic surface (concrete, plastic or wood surface; litter). The data were transformed into a Likert scale, 1–5 from minimum to maximum, proportional to the absolute values.

The individuals sampled were identified *de visu* with taxonomic guides first, then species identification was confirmed from DNA barcoding as detailed in Ardura *et al*.^5^. In summary, DNA was extracted from muscle tissue and a fragment within the mitochondrial Cytochrome oxidase I gene (COI) was PCR amplified using Geller *et al*.^43^ primers, and sequenced. Some individuals were double-checked with a second marker, the 16S rRNA gene with the primers described by Palumbi^44^, to confirm the species when identification using COI was not sufficiently accurate (99% match, at least 450 nucleotides coverage). The sequences were compared with international databases such as the BOLD system for COI (http://www.boldsystems.org/) and the program BLAST within NCBI for 16S rRNA gene sequences (http://www.ncbi.nlm.nih.gov/) for identifying the species.

The native or exotic status of the species found in a site was assigned based on the native distribution of each species (World Register of Marine Species, www.marinespecies.org; Encyclopedia of Life, available from http://www.eol.org, accessed September 2015), and in the case of Vancouver Island also on the list of exotic marine fauna from British Columbia that was found online in the Electronic Atlas of Wildlife of British Columbia (http://ibis.geog.ubc.ca/biodiversity/efauna/MarineInvasiveSpecies.html, accessed September 2015).

Two measures of biodiversity were considered: species richness and Simpson’s index (this measure also considers evenness). They were calculated for each site using the software PRIMER v.6 45.

### Analytical framework

#### A-Quantification of vectors

The vectors considered in this study, maritime traffic and aquaculture, were recognized facilitators of marine invasions.

1-Maritime traffic was quantified as explained in Ardura *et al*.^5^ Briefly, the number of boats visible from each sampling location was counted for half an hour on three different days to quantify maritime traffic. It was a proxy of local shipping intensity. In addition the distance to ports (in km along the coast) was also included in the analysis. Maritime traffic reflected local movements of ships, generally small vessels and boats than could stop everywhere. Ports received fishing boats, ferries and other vessels that were connected with big ports. They were main entry points of exotic biota and local maritime traffic contributed to its dispersal across the region.

2-Aquaculture as a vector of biological invasions was more difficult to quantify because, in addition to the number of farms, the different species (that potentially occupied different niches) should also be taken into account. The size of farms (volume of production in a zone) should be also considered. Although farms of exotic species were obvious vectors due to potential escapes, farms of native species would also favor NIS if they contributed to habitat degradation. On Vancouver Island, in addition to the three mussel species reported in Crego-Prieto *et al*.^6^ there was *Crassostrea gigas* farming in Fanny Bay and Nanoose Bay, and *Ruditapes philippinarum* aquaculture on Cortes Island and Baynes Sound (near Fanny Bay). In Moorea Island there were only two sites with aquaculture, one farm of imported *Penaeus stylirostris* in Opunahu Bay and one small touristic exhibition farm of the native pearl oyster, *Pinctada margaritifera*, in Tiki (Fig. 1).

Because the sites were very different regarding shellfish farming, we used the following formula (1) to standardize quantification of aquaculture as a NIS vector:


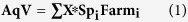


where AqV was Aquaculture value as a vector; X was a weight factor that depended on the cultivated species, 1 for native, 2 for exotic and 3 for recognized invader from the IUCN list; Sp_i_ was the species *i*; Farm_i_ was the number of farms of the species *i* within a 10 km distance of the sampling site.

After measuring each vector in each site, data were transformed into a Likert scale to normalize data for statistical treatment. We used a 0–5 scale with decimals from the minimum to the maximum expression of each factor in the 30 study sites throughout Moorea and Vancouver islands.

#### B-Protection scheme

“Protection” meant banning anthropogenic actions for exploiting the territory such as fishing, building new houses, forestry, aquaculture, installing ferry lines etc. The term “protection” was objectively measured here from the number of exploitation activities allowed in a site (with negative sign, the more activities the lower score, 1–5 from more to less activities). For the **protective status**, there were three different intensities on each island. MPAs of Moorea and National Parks of Vancouver Island could be considered level one since the restrictions to human activities were similar in the two cases, with aquaculture, harvesting, urbanization and modifications to the natural habitat strictly regulated. They thus represented the highest level of protection. The second level of protection corresponded to ecological reserves and fisheries regulated areas in Vancouver Island and Moorea respectively, with many exploitation activities banned or regulated. Regulated fisheries in Moorea (less restrictive than in Vancouver) and regional parks in Vancouver provided some restrictions to human activities and were considered as the third level. Finally, zones of free use with no protection were the basic standard level. We assigned to each level a value of 5, 3, 2 and 1 respectively, from maximum to minimum (no protection). The gap between the maximum and the next level of protection was justified by the number of uses allowed in the second level of protection, comparatively much higher than in MPAs and National Parks. At our smallest scale, this value corresponded to the protection status of the area where the sampling point was located.

The density of protected areas (as a proxy of connectivity) was also considered, as a weighted average. At a medium scale we quantified the length of coast protected within 10 km from each sampling point. The value was calculated as the number of kilometers protected multiplied by the weight of the corresponding protection figure as outlined above and divided by the total number of kilometers (in this case 10). For example, if in 10 km of coast there was 1 km fully protected (level 5), 3 km mid-protected (level 3), 2 km of little protection (level 2) and the rest with no special measures of protection (level 1), the global score for these 10 km was 0.1 × 5 + 0.3 × 3 + 0.2 × 2 + 0.4 × 1 = 2.2. At a larger scale we considered 60 km of coast and performed the same calculation. For Moorea Island it corresponded to the whole island perimeter. For Vancouver Island it represented the 60 km of coast surrounding each sampling point in the west or east coast. In addition to the size, as seen above the duration of the protective status was important^22^. This was incorporated as the time (age) of the protected areas. Average scores per kilometer were given for comparable (not nested) values for small, medium and large scale.

The formula (2) applied for quantifying the level of protection (per km) in an area (medium and large scale) was:


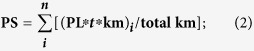


where PS was protection status of an area, PL was the protective level (from 1 to 5 as explained above) of status *i*, *t* was the duration of the protection status, km was the number of kilometers. The units were therefore protection*time (final units being standardized protection years), protection being measured from the number of exploitation activities banned in a place (see above).

#### Mathematical models

The NIS vectors and marine protection schemes were expected to act in opposite directions on NIS. If protective spatial status conferred protection against NIS (impeding their settlement by improving native biodiversity and/or by reducing habitat disturbances), it should minimize the impact of NIS vectors. Since other habitat and community characteristics and not only NIS vectors may concur in the settlement of NIS, we considered the following variables (measured as explained above): proportion of NIS; number of native species; spatial status (protective status) at different scales; distance to ports; local maritime traffic; aquaculture; substrate artificiality; exposure to waves; pollution; freshwater proximity; algae coverage. Proportion of NIS was chosen instead of direct counts because sampling was approximately proportional to the abundance of each species. Normality in residuals was checked using the Shapiro-Wilk test.

In an initial analysis of the dataset we conducted pairwise correlations and a principal component analysis (PCA) with PAST software^46^ version 3.11 for Mac OSX 10.8 and later. The objective was to identify possible internal correlations between variables as well as possible outlier samples that could interfere in the models. The correlation option and 1 000 bootstraps were employed. Outliers were removed from further analysis. When pairwise correlations were performed on the whole dataset Bonferroni correction for multiple comparisons was applied, that was 0.0042 for 12 correlations (13 variables involved) and P = 0.05 cutoff.

We considered different models (multivariate and bivariate) and implemented them using PAST. If the results obtained from the two different approaches were consistently similar, we could assume reasonably that the conclusions were robust. For the multivariate analysis we used Multiple Linear Regression (MLR), with one dependent (NIS proportion) and several independent variables (the rest of the variables considered). An overall ANOVA-type significance test of multivariate regression was calculated, based on SSR (regression sum of squares) and SSE the error (residuals) sum of squares, with n samples and k independent variables as F  =  (SSR/k)/[SSE/(n-k-1)].

For bivariate analyses we used a Generalized Linear Model (GLM) for single explanatory variables, assuming a normal distribution with NIS as the dependent variable and the other variables, in pairs, as independent variables. This was equivalent to Ordinary Least Squares Linear Regression. We also used normal distribution and log link functions to fit the logarithmic function. We tested for autocorrelations using a Durbin-Watson statistic (based on residuals from OLS regression), and for homoskedasticity with Breusch-Pagan tests.

#### Model tests in partial datasets

We further tested the models using partial datasets, considering different regional combinations of samples. The rationale of this approach was that the study was correlational but the two islands were very different in terms of both protective status and relative impact of the different NIS vectors. These differences could induce analytical artifacts when the two islands were combined. If the same variables were significant in the models whatever the regional combination analyzed, their correlation with NIS proportion (in a negative or positive way) could be repeatable in different regions around the world. Thus we tested the MLR and GLM models in the following groups of samples: [east Vancouver Island + Moorea], [west Vancouver Island + Moorea], Vancouver Island alone, Moorea alone. Consistent results would allow us to be reasonably sure that the conclusions were robust and not due to analytical artifacts produced from the combination of very different datasets.

## Additional Information

**How to cite this article**: Ardura, A. *et al*. Rate of biological invasions is lower in coastal marine protected areas. *Sci. Rep*. **6**, 33013; doi: 10.1038/srep33013 (2016).

## Supplementary Material

Supplementary Information

## Figures and Tables

**Table 1:** Characteristics of the samples analyzed in this study.

	Protective status			Site characteristics								Species		Diversity	
	Status	Status-10 km	Status-60 km	Port	Freshwater	Pollution	Algae	Waves	Traffic	Substrate	Aquaculture	NIS	Native	SR	Simpson
M-PP	1	0.252	1.575	0.2	0.1	0.03	0.1	4	4	5	0	0.4	3	0.8764	0.5195
M-Pa	1	0.5355	1.575	0.3	0.05	0.1	0.4	3	3	2	0	0.17	5	1.095	0.7761
M-Th	1	0.567	1.575	0.5	1	0.05	0.3	3	2	3	0	0.2	4	0.8784	0.5991
M-Fa	1	0.252	1.575	2	0.1	1	0.2	3	1	3	0	0.17	5	1.117	0.6157
M-Vn	1	0.189	1.575	0.2	0.5	0.3	0.3	3	3	5	0	0.2	4	0.9294	0.7149
M-Vr	1	0.0945	1.575	0	0.8	0.8	0.1	4	5	5	0	0.17	10	2.34	0.7248
M-Ti	5	0.315	1.575	6	1	0.02	0.1	2	3	5	0.079365079	0	1	0	0
M-At	3	0.36225	1.575	0.2	0.8	1	0.1	5	1	1	0	0	3	0.4402	0.2743
M-Hu	5	0.7875	1.575	3	1	0.5	0.1	2	2	5	0	0	2	0.2191	0.02083
M-Ha	3	0.3465	1.575	3	0.2	0.5	0.1	3	1	1	0	0	2	0.2191	0.4342
M-Mh	2	0.315	1.575	0.2	0.02	0.1	0.1	4	3	4	0	0	4	0.6635	0.4872
M-Te	5	0.36225	1.575	0.3	1	0.1	0.1	4	3	5	0	0	3	0.4382	0.534
M-Ma	5	0.42525	1.575	0.3	1	0.02	0.3	5	2	5	0	0	3	0.4456	0.4668
M-EB	2	0.4095	1.575	2	1	0.2	0.5	3	4	4	0	0	9	1.842	0.6364
M-Op	3	0.441	1.575	0.2	0.3	0.1	0.1	1	1	1	0.158730159	0	5	0.8764	0.5934
M-Af	3	0.3465	1.575	0.05	0.1	0.03	0.1	5	3	4	0	0	4	0.6573	0.6287
V-CI	1	0.0282	0.705	2.2	0.5	0.02	0.7	3	5	3	5	0.5	1	0.2612	0.1511
V-Cr	1	0.0141	0.705	1.6	0	0.1	0.9	1	4	3	1.19047619	0.4	3	0.9894	0.4974
V-FB	1	0.0141	0.705	2.9	0	0.8	0.6	2	2	4	3.571428571	0.6	4	2.031	0.7388
V-NB	1	0.02115	0.705	2.3	1	0.5	0.1	2	3	4	1.904761905	0.29	5	1.347	0.7753
V-PI	1	0.0141	0.705	4.2	0	0.5	0.2	2	4	1	0.476190476	0.125	7	1.642	0.6215
V-Ld	3	0.0846	0.705	6.8	0.9	0.1	0.9	1	2	3	1.19047619	0	3	0.4692	0.6374
V-Sd	2	0.0141	0.705	0.34	0	0.5	0.3	1	4	2	0.476190476	0	8	1.683	0.5229
V-Vi	3	0.0846	0.705	1.1	0	0.1	0.8	4	3	4	0	0	6	1.103	0.653
V-Sk	1	0.6	5	0	0	0.1	0.5	1	5	4	0	0.111	8	1.791	0.6289
V-CB	3	1	5	30	1	0.5	0.1	5	2	2	0	0	8	1.571	0.691
V-Bf	5	5	5	0	0	0.1	0.9	3	2	3	1.666666667	0	6	1.106	0.7275
V-PA	1	0.2	5	0	0.1	0.3	0.9	1	1	5	0	0	2	0.2556	0.2423
V-SB	1	0.1	5	9.3	0.53	0.5	0.5	5	2	2	1.666666667	0.1	9	2.015	0.7781
V-LB	1	0.9	5	18.7	0.95	1	0.5	5	1	1	0	0.111	8	1.626	0.4825

Protective status: for 10 km and 60 km protective status corresponds to the average level of spatial protection per km (on a Likert scale) in 10 and 60 km, respectively, around the sampling site. Site characteristics: Distance in km to the nearest port (Port), freshwater discharge (Freshwater) and pollution source (Pollution); Algae, algae coverage (as proportion of surface covered); exposure to waves (Waves), maritime traffic (Traffic), substrate artificiality (Substrate) and aquaculture are Likert-scaled 1 to 5 from minimum to maximum intensity. Species: NIS, proportion of Non-Indigenous Species over the total number of species; Native, number of native species. Diversity: SR, species richness; Simpson, Simpson’s diversity index. Site acronyms (M for Moorea and V for Vancouver): PP, Pao-Pao; Pa, Papetoai; Th, Tiahura; Fa, Faarehau; Vn, Vai’ane; Vr, Vai’are; Ti, Tiki; At, Atiha; Hu, Hauru; Mh, Maharepa, Te, Temae; Ma, Maatea; EB, Entre-2−Baies; Op, Opunohu; Af, Afareitou; CI, Cortes Island; Cr, Crofton; FB, Fanny Bay; NB, Nanoose Bay; PI, Portland Island; Ld, Ladysmith; Sd, Sydney; Vi, Victoria; Sk, Sooke; CB, China Beach; Bf, Bamfield; PA, Port Alberni; SB, Salmon Beach; LB, Long Beach.

**Table 2:** Statistical analysis.

	PC 1	PC 2	PC 3	PC 4
Eigenvalue	30.1 (11.7–37.4)	22.6 (9.0‒27.7)	18.2 (16.3–25.4)	13.5 (4.9‒19.5)
Variance	23.2%	17.4%	14.0%	10.4%
NIS	−**0.40975**	0.28584	−0.09208	0.22396
Status ‒ local	0.25413	−**0.48867**	0.15712	0.18044
Status ‒ 10 km	0.21192	−0.044928	**0.55451**	−0.0046641
Status ‒ 60 km	0.32992	0.21785	0.37577	−0.1567
Distance to port	**0.33782**	0.30027	−0.033574	0.16979
Wave exposure	0.30499	0.034612	−0.19893	−0.015825
Distance to freshwater	0.25111	−0.15278	−0.27203	0.23344
Algae coverage	−0.1873	0.15759	**0.5384**	0.086613
Distance to pollution	0.1906	**0.39351**	‒0.27569	0.18875
Local maritime traffic	−0.33105	0.0024585	‒0.081155	‒**0.50518**
Substrate artificiality	‒0.17276	‒0.3697	‒0.026772	‒0.18746
Aquaculture	−0.31618	0.22915	0.17362	0.42082
Native species	0.18095	0.38267	‒0.011198	‒**0.54641**

Principal Component Analysis showing the principal components (PC) and loadings of the variables considered in this study. Eigenvalues are given with 2.5% and 97.5% limits in parenthesis. The two variables with higher load in each component are highlighted in bold.

**Table 3:** Models applied to the dataset.

	Multiple Linear Regression									
	Coefficient	SE	t	P‒value	R^2^	Generalized Linear Model				
Dependent variable (NIS)	0.24	0.109	2.215	0.042		Phi	Slope	Intercept	G	P (slope = 0)
**Status** ‒ **local**	−**0.079**	−**0.017**	−**4.528**	**0.00029**	**0.361**	**0.019**	−**0.068**	**0.2755**	**15.002**	**0.0001**
Status‒ 10 km	0.046	0.032	1.386	0.185	0.053	0.027	−0.045	0.139	1.734	0.187
Status ‒ 60 km	−0.032	0.018	−1.704	0.109	0.076	0.027	−0.031	0.181	2.519	0.112
Distance to port	0.002	0.01	1.199	0.844	0.001	0.028	−0.003	0.127	0.323	0.569
Wave exposure	0.003	0.015	0.185	0.855	0.015	0.028	−0.018	0.172	0.665	0.414
Distance to freshwater	−0.029	0.055	−0.546	0.592	0.036	0.027	−0.082	0.156	1.337	0.247
Algae coverage	−0.001	0.001	−0.864	0.401	0.031	0.028	0.001	0.078	1.091	0.296
Distance to pollution	0.035	0.081	0.433	0.671	0.013	0.029	0.044	0.103	0.211	0.645
Local maritime traffic	0.01	0.023	0.427	0.675	0.115	0.026	0.045	−0.004	3.678	0.055
Substrate artificiality	0.033	0.017	1.938	0.072	0.007	0.029	0.012	0.078	0.312	0.576
**Aquaculture**	**0.071**	**0.022**	**3.214**	**0.005**	**0.451**	**0.017**	**0.095**	**0.063**	**21.316**	**<<0.0001**
Native species	−0.017	0.011	−1.496	0.155	0.015	0.028	−0.009	0.166	0.644	0.422

Results of the Multiple Linear Regression and Generalized Linear models for each considered variable, after excluding outlier samples.

Significant variables are marked in bold. NIS, proportion of Non Indigenous Species. SE, standard error. Phi, dispersion phi‒value.

**Table 4:** Application of the Multiple Linear Regression and Generalized Linear models to different regional combinations of samples, with NIS (Non Indigenous Species) proportion as dependent variable.

	Multiple Linear Regression					Generalized Linear Model				
	ANOVA	Variable	Coefficient (SE)	t	P-value	Phi	Slope	Intercept	G	P (slope = 0)
Vancouver W + Moorea (N = 22)	F = 2.37, P = 0.100	Status‒local	−0.091 (0.002)	−4.159	0.002	0.007	−0.043	0.179	14.656	0.0001
Vancouver E + Moorea (N = 24)	F = 6.085, P = 0.002	Status‒local	−0.076 (0.019)	−3.795	0.003	0.019	−0.082	0.322	17.729	0.00003
		Aquaculture	0.062 (0.027)	2.329	0.039	0.016	0.105	0.073	24.871	<0.00001
		Substrate	0.042 (0.017)	2.423	0.034	0.034	0.014	0.087	0.271	0.603
Vancouver Island (N = 14)	F = 19.9, P = 0.055	Status‒local	−0.223 (0.014)	−15.551	0.041	0.033	−0.086	0.314	4.608	0.032
		Aquaculture	0.079 (0.005)	15.639	0.040	0.017	0.108	0.027	19.905	<0.00001
Moorea (N = 16)	F = 3.169, P = 0.049	Status‒local	−1.735 (0.717)	−2.420	0.034	1.791	−0.222	3.189	8.197	0.004

The variables with statistical significance in at least one of the models are presented. Vancouver W and Vancouver E are the west and east coast of Vancouver Island respectively. SE, standard error. Phi, dispersion phi‒value.
