# Supplementary material for: Rate of biological invasions is lower in coastal marine protected areas
Source: Sci Rep. 2016 Sep 9;6:33013. doi: 10.1038/srep33013 (PMC5016778; doi:10.1038/srep33013)
Supplement: Supplementary Information [file srep33013-s1.doc]

**Manuscript title:**

**Rate of biological invasions is lower in coastal marine protected areas.**

**List of authors:** Alba Ardura^1^, Francis Juanes^2^, Serge Planes^1^, Eva Garcia-Vazquez^3^

**Affiliations:**

1: Laboratoire d’Excellence « CORAIL », USR3278-CRIOBE-CNRS-EPHE-UPVD, Université de Perpignan-CBETM, 58 rue Paul Alduy, 66860 Perpignan Cedex, France

2: Department of Biology, University of Victoria, Victoria, BC, Canada V8W 3N5

3: Department of Functional Biology, University of Oviedo. C/ Julian Claveria s/n. 33006-Oviedo, Spain.

Corresponding author: Eva Garcia-Vazquez [egv@uniovi.es](mailto:egv@uniovi.es)

**Supplementary Table 1**. Inventory of mollusk species found in the 14 sampling sites of Vancouver Island. Area sampled at each site: 200m^2^. N: total number of individuals. NIS: number of individuals of non-indigenous species. N Spp: total number of species in a site. NIS are marked in bold in the species list.

| Species | Location | Fanny Bay | Nanoose Bay | Crofton | Lady Smith | Sidney | Victoria | Cortes Island | Portland Island | Sooke | China Beach | Port Alberni | Bamfield | Salmon Beach | Long Beach | **N** |
| --- | --- | --- | --- | --- | --- | --- | --- | --- | --- | --- | --- | --- | --- | --- | --- | --- |
| ***Batillaria atramentaria*** | | 13 | 0 | 0 | 0 | 0 | 0 | 0 | 0 | 0 | 0 | 0 | 0 | 0 | 0 | **13** |
| *Clinocardium nuttallii* | | 0 | 0 | 0 | 0 | 0 | 0 | 0 | 0 | 1 | 0 | 0 | 0 | 0 | 0 | **1** |
| ***Crassostrea gigas*** | | 9 | 4 | 3 | 0 | 0 | 0 | 0 | 0 | 1 | 0 | 0 | 0 | 0 | 0 | **17** |
| *Cyanoplax dentiens* | | 0 | 0 | 0 | 0 | 0 | 1 | 0 | 0 | 0 | 0 | 0 | 0 | 0 | 0 | **1** |
| *Doris montereyensis* | | 0 | 0 | 0 | 0 | 2 | 0 | 0 | 0 | 0 | 0 | 0 | 0 | 0 | 0 | **2** |
| *Katharina tunicata* | | 0 | 0 | 0 | 0 | 0 | 1 | 0 | 0 | 0 | 0 | 0 | 0 | 0 | 0 | **1** |
| *Leukoma staminea* | | 0 | 0 | 0 | 0 | 1 | 0 | 0 | 0 | 0 | 0 | 0 | 0 | 0 | 0 | **1** |
| *Lirabuccinum dirum* | | 0 | 0 | 0 | 0 | 0 | 0 | 0 | 0 | 0 | 0 | 0 | 8 | 3 | 0 | **11** |
| *Pomaulax gibberosus* | | 0 | 0 | 0 | 0 | 0 | 0 | 0 | 0 | 0 | 0 | 0 | 0 | 3 | 0 | **3** |
| *Littorina plena* | | 0 | 0 | 4 | 0 | 0 | 0 | 0 | 0 | 0 | 0 | 0 | 0 | 0 | 0 | **4** |
| *Littorina scutulata* | | 0 | 10 | 0 | 0 | 0 | 0 | 0 | 0 | 0 | 0 | 0 | 0 | 0 | 0 | **10** |
| *Littorina sitkana* | | 0 | 1 | 0 | 0 | 0 | 10 | 0 | 9 | 10 | 2 | 0 | 0 | 0 | 0 | **32** |
| *Lottia digitalis* | | 0 | 0 | 0 | 0 | 0 | 0 | 0 | 0 | 0 | 0 | 0 | 4 | 0 | 1 | **5** |
| *Lottia paradigitalis* | | 0 | 11 | 0 | 0 | 0 | 0 | 0 | 0 | 2 | 10 | 0 | 0 | 4 | 0 | **27** |
| *Lottia pelta* |  | 3 | 0 | 0 | 2 | 6 | 3 | 0 | 0 | 16 | 6 | 0 | 4 | 9 | 4 | **53** |
| *Lottia persona* | | 9 | 13 | 0 | 0 | 0 | 0 | 0 | 3 | 4 | 6 | 0 | 0 | 0 | 0 | **35** |
| *Lottia scutum* | | 0 | 0 | 2 | 19 | 2 | 34 | 0 | 8 | 0 | 0 | 0 | 4 | 0 | 1 | **70** |
| *Macoma balthica* | | 0 | 0 | 0 | 0 | 0 | 0 | 0 | 0 | 0 | 0 | 2 | 0 | 0 | 0 | **2** |
| *Mytilus californianus* | | 0 | 0 | 0 | 0 | 0 | 0 | 0 | 0 | 0 | 48 | 0 | 0 | 23 | 55 | **126** |
| ***Mytilus edulis*** | | 1 | 0 | 0 | 0 | 0 | 0 | 0 | 0 | 0 | 0 | 0 | 0 | 0 | 0 | **1** |
| ***Mytilus galloprovincialis*** | | 3 | 0 | 0 | 0 | 0 | 0 | 1 | 0 | 0 | 0 | 0 | 0 | 0 | 0 | **4** |
| *Mytilus trossulus* | | 39 | 42 | 45 | 50 | 46 | 44 | 45 | 44 | 49 | 1 | 48 | 49 | 25 | 1 | **528** |
| *Nucella lamellosa* | | 2 | 0 | 0 | 0 | 4 | 0 | 0 | 2 | 3 | 11 | 0 | 0 | 0 | 0 | **22** |
| ***Nucella lapillus*** | | 0 | 0 | 0 | 0 | 0 | 0 | 0 | 3 | 0 | 0 | 0 | 0 | 0 | 0 | **3** |
| *Nucella ostrina* | | 0 | 0 | 0 | 0 | 0 | 0 | 0 | 1 | 0 | 0 | 0 | 23 | 0 | 2 | **26** |
| ***Nuttalia obscurata/olivacea*** | | 1 | 0 | 0 | 0 | 0 | 0 | 0 | 0 | 0 | 0 | 0 | 0 | 1 | 0 | **2** |
| *Onchidoris muricata* | | 0 | 0 | 0 | 0 | 1 | 0 | 0 | 0 | 0 | 0 | 0 | 0 | 0 | 0 | **1** |
| *Protothaca staminea* | | 0 | 0 | 0 | 0 | 0 | 0 | 0 | 0 | 0 | 0 | 0 | 0 | 1 | 0 | **1** |
| ***Ruditapes philippinarum*** | | 4 | 5 | 3 | 0 | 0 | 0 | 0 | 0 | 0 | 0 | 0 | 0 | 0 | 1 | **13** |
| *Tectura persona* | | 0 | 0 | 0 | 0 | 2 | 0 | 0 | 1 | 0 | 2 | 0 | 0 | 0 | 3 | **8** |
| *Tegula funebralis* | | 0 | 0 | 0 | 0 | 0 | 0 | 0 | 0 | 1 | 0 | 0 | 0 | 17 | 7 | **25** |
| *Tresus capax* |  | 0 | 0 | 0 | 0 | 0 | 0 | 0 | 0 | 0 | 0 | 0 | 0 | 1 | 0 | **1** |
|  | **N** | **84** | **86** | **57** | **71** | **64** | **93** | **46** | **71** | **87** | **86** | **50** | **92** | **87** | **75** | **1049** |
|  | NIS | 31 | 9 | 6 | 0 | 0 | 0 | 1 | 3 | 1 | 0 | 0 | 0 | 1 | 1 | 53 |
|  | **N Spp** | **10** | **7** | **5** | **3** | **8** | **6** | **2** | **8** | **9** | **8** | **2** | **6** | **10** | **9** | **32** |
